# Primary Extramammary Paget’s Disease Following Previously Treated Secondary Extramammary Paget’s Disease Over the Scrotum: A Rare Case Successfully Treated With Mohs Micrographic Surgery

**DOI:** 10.7759/cureus.63160

**Published:** 2024-06-25

**Authors:** Kritin K Verma, Nabeel Ahmad, Daniel P Friedmann, Brian L Ransdell, Michelle Tarbox

**Affiliations:** 1 Medicine, Texas Tech University Health Sciences Center, Lubbock, USA; 2 Dermatology, University of Houston, Houston, USA; 3 Westlake Dermatology Clinical Research Center, Westlake Dermatology and Cosmetic Surgery, Austin, USA; 4 Dermatology, Longhorn Dermatology, Austin, USA; 5 Dermatology, Texas Tech University Health Sciences Center, Lubbock, USA

**Keywords:** histopathology (hp), mohs micrographic surgery, redevelopment, secondary extramammary paget’s disease, primary extramammary paget’s disease

## Abstract

Extramammary Paget's disease (EMPD) is a rare cutaneous neoplasm that can be classified as either primary or secondary, depending on the presence or absence of an associated internal malignancy. Primary EMPD arises as an intraepithelial adenocarcinoma, while secondary EMPD results from the extension of an underlying visceral malignancy. This case report presents a unique instance of primary EMPD developing 10 years after a diagnosis of secondary EMPD in the same anatomical location, a phenomenon not previously documented in the literature. The patient, initially treated for secondary EMPD with wide local excision, later developed primary EMPD, as confirmed through histopathological and immunohistochemical analysis. This rare occurrence raises questions about the potential mechanisms, including field cancerization, persistent risk factors, or a coincidental event. The case underscores the importance of long-term follow-up and surveillance for EMPD patients. Mohs micrographic surgery remains the gold standard for treating EMPD due to its high precision in margin control and lower recurrence rates compared to conventional surgical methods. This case highlights the need for meticulous diagnostic approaches and continuous monitoring to manage and understand the complexities of EMPD effectively.

## Introduction

Extramammary Paget’s disease (EMPD) is an intra-epithelial malignancy that occurs within apocrine gland-bearing skin, such as the vulva, scrotum, penis, perineum, or axillae [1]. It typically presents clinically as demarcated erosive, ulcerated, or scaly plaques [1]. Primary EMPD originates in the epidermis or apocrine sweat glands as a primary intraepithelial adenocarcinoma, whereas secondary EMPD represents extension or metastasis from an underlying internal malignancy, most commonly from the genitourinary or gastrointestinal tract [1,2]. Toker cells, which are epithelial cells found in body areas abundant in apocrine glands, have been suggested as a possible cause of primary EMPD [1]. Secondary EMPD constitutes a direct epidermal extension or metastatic epidermal spread of an underlying adnexal or visceral (e.g., urothelial or anorectal) adenocarcinoma [1,2].

We describe what we believe to be the first case of primary EMPD of the left periscrotal inguinal crease that occurred years after the diagnosis of a secondary EMPD in the same region. Our patient was successfully treated with Mohs micrographic surgery (MMS) for their primary EMPD, 10 years following wide local excision (WLE) of an initial diagnosis of secondary EMPD in the same area.

## Case presentation

A 75-year-old male patient presented to our clinic with hypertrophic, erythematous scarred nodules of the left inguinal crease approximating the scrotum. Ten years earlier, he had a WLE performed at an outside clinic for secondary EMPD of the same region, which was then thought to be associated with a recent diagnosis of bladder cancer (grade 2 Ta urothelial cell carcinoma (UCC) or multiple colon polyps). The patient remained tumor-free for five years, at which point he was treated for recurrent grade 3 Ta UCC and high-grade prostatic intraepithelial neoplasia. He presented to our clinic five years after that therapy with a progressively enlarging lesion in the left groin that had begun to spread periscrotally.

**Figure 1:**
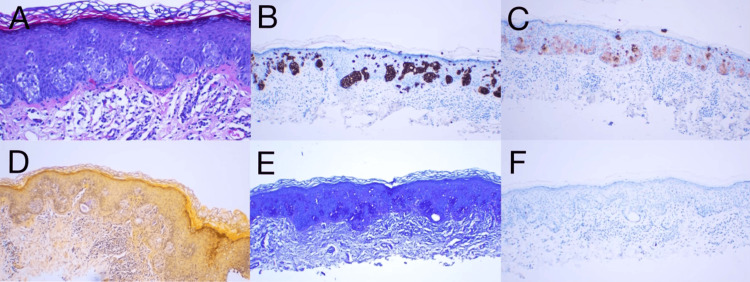
Histopathology of primary EMPD. (A) H&E stain (200x) demonstrated orthokeratosis overlying a mildly thickened epidermis with a large collection of pale-staining cells, compressing the basal cell layer. (B) Immunohistochemical staining positive for CK7 (100x). (C) Immunohistochemical staining positive for CEA (100x). (D) Immunohistochemical staining positive for mucicarmine (100x). (E) Immunohistochemical staining positive for PAS (100x). (F) Immunohistochemical staining negative for CK20 (100x) CEA: carcinoembryonic antigen; CK: cytokeratin; EMPD: extramammary Paget’s disease; H&E: hematoxylin and eosin; PAS: periodic acid-Schiff

Histopathologic evaluation of the nodule via incisional biopsy demonstrated orthokeratosis overlying a mildly thickened epidermis with a large collection of pale-staining cells, which appear to compress the basal cell layer (Figure 1A). Observed was a mild inflammatory infiltrate within the dermis. Lesional cells highlight strongly and diffusely with CK7 (Figure 1B) and to a lesser extent with carcinoembryonic antigen (Figure 1C). Mucicarmine stain shows mucin within the epidermis (Figure 1D), and periodic acid-Schiff slightly enhances lesional cells (Figure 1E). CK20 staining was negative (Figure 1F), indicative of primary EMPD. A CT scan, cystoscopy, and colonoscopy were negative for occult malignancy.

**Figure 2:**
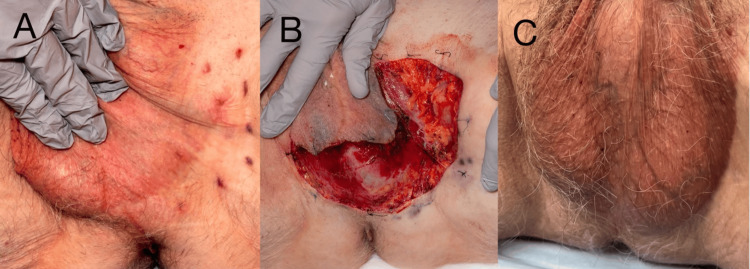
(A) EMPD of the left inguinal crease refractory to brachytherapy. (B) Inguinal region defect following tumor extirpation with MMS. (C) One-year postsurgical result demonstrating excellent cosmesis EMPD: extramammary Paget’s disease; MMS: Mohs micrographic surgery

Brachytherapy was performed with clinical benefit but with residual tumors evident on pathology (Figure 2A). MMS was then performed in the left inguinal crease, extending into the periphery of the scrotum (Figure 2B). The patient had an uneventful postoperative course and remains without evidence of recurrence more than one year later (Figure 2C). He continues to be closely followed for potential underlying occult gastrointestinal or prostate malignancies.

## Discussion

EMPD recurrence is common within the first two years postoperatively, with the recurrent primary disease having a good prognosis [3]. Invasive or metastatic primary EMPD and recurrent secondary EMPD are associated with a poorer prognosis, even after an adequate response to first-line chemotherapy [3]. Nevertheless, EMPD secondary to UCC may present the malignancy asynchronously, with the cutaneous neoplasm occurring years before or after the detection of the malignancy [4]. The diagnosis of primary vs. secondary EMPD in the setting of an occult malignancy, as was suspected in this patient, may be challenging, as the clinical presentation may be equivalent. Immunohistochemistry may be useful in discriminating primary EMPD (HER-2/neu positive, CDX2 negative) from EMPD secondary to anorectal adenocarcinoma (HER-2/neu negative, CDX2 positive), but not EMPD secondary to urothelial or prostatic malignancy [5].

The treatment of primary vs. secondary EMPD is understandably different. While primary EMPD rests on surgical excision, secondary EMPD requires management of the underlying malignancy in conjunction with surgical therapy of the cutaneous tumor [6]. MMS may be superior to WLE for the surgical management of EMPD, especially in more aggressive forms of EMPD [6]. A systematic review and meta-analysis demonstrated a 7.3% local recurrence rate after MMS compared to 26.3% after WLE [7]. Although regular MMS led to complete clearance of EMPD in this case, modified slow MMS may provide better clinical outcomes [8,9]. Adjuvant immunotherapy with topical imiquimod 5%, brachytherapy, photodynamic therapy, or external beam radiotherapy may decrease the risk of EMPD recurrence following definitive surgical therapy [10].

We describe a case of recurrent EMPD, although the initial presentation was of secondary EMPD (CK20 positive secondary to UCC) a decade prior to the redeveloped lesion, indicative of primary EMPD (CK20 negative) located at the same left periscrotal inguinal area, which is an extremely uncommon and unusual phenomenon [1]. Primary EMPD develops as an intraepithelial tumor of the epidermis, whereas secondary EMPD is caused by the epidermotropic spread of malignant cells or direct extension from an underlying internal neoplasm [1,2]. One possible explanation for such a scenario is a coincidental occurrence, in which the main EMPD develops independently of the prior secondary EMPD [11]. Furthermore, the concept of field cancerization implies that the entire area of skin or mucosa is predisposed to developing multiple independent primary tumors due to genetic or environmental factors, potentially leading to the development of a separate primary EMPD years after the initial secondary EMPD [11,12].

It is crucial to perform histopathological and immunohistochemical analyses to accurately differentiate between primary and secondary EMPD [11]. The distinction is vital as it impacts the treatment strategy and prognosis, with primary EMPD generally having a better prognosis compared to secondary EMPD, which is associated with the underlying malignancy [12]. Close surveillance following surgical management (MMS) will be essential in monitoring for an underlying malignancy and lesion recurrence, especially for complex cases of primary EMPD development following secondary EMPD.

## Conclusions

This case report presents a unique and unprecedented instance of primary EMPD developing 10 years after a diagnosis of secondary EMPD in the same anatomical location, specifically the left periscrotal inguinal crease. The initial secondary EMPD was associated with UCC and was treated with WLE. A decade later, the patient developed primary EMPD, confirmed through histopathological and immunohistochemical analysis, which highlighted the absence of CK20 staining, indicative of primary EMPD. This rare occurrence raises important questions about potential mechanisms, including the possibility of field cancerization, persistent risk factors, or a coincidental event. The case underscores the critical importance of long-term follow-up and surveillance in EMPD patients, as well as the need for meticulous diagnostic approaches to accurately differentiate between primary and secondary EMPD. MMS was successfully employed in this case, demonstrating its efficacy as the gold standard for treating EMPD due to its high precision in margin control and lower recurrence rates compared to conventional surgical methods. This case highlights the complexities of EMPD and the necessity for continuous monitoring and advanced surgical techniques to manage and understand this rare disease effectively.
